# Thirty-fifth Annual Meeting of the Illinois State Medical Society

**Published:** 1885-06

**Authors:** 


### THIRTY-FIFTH ANNUAL MEETING OF THE ILLINOIS STATE MEDICAL SOCIETY, SPRINGFIELD, ILL., MAY 19TH, 20TH AND 21ST, 1885.

### *First Day—Tuesday, May 19th.*

#### MORNING SESSION.

The Society was called to order at 10 A. M., by the President, Dr. D. S. BOOTH, of Sparta. After prayer by Rev. F. H. MUSGROVE, of Springfield, the address of welcome was delivered by his Excellency, GENERAL RICHARD J. OGLESBY, GOVERNOR OF THE STATE OF ILLINOIS. Governor Oglesby's remarks were brief and felicitous.

He desired to welcome the members of the Illinois State Medical Society to the Capital, as gentlemen, citizens and physicians. He congratulated the people of the State, that so many representative medical men could be found willing to leave their homes, with absolute unselfishness, to consult together for the public good. To the physicians, more than to any other body of men, is entrusted the health, prosperity and life of the people. The fact of this annual convention, attended by so many medical men, who had at considerable sacrifice forsaken their daily avocations, was self-evident proof of their devotion to the profession of medicine and surgery and to the trust reposed in them.

He recognized in the science of medicine and surgery a field of infinite possibility as regards development. It was the function of these annual meetings to lift the subject above illiberal and envious criticism, and establish it upon a more enduring basis than that of empiricism. It was true that the science of medicine and surgery was not as exact as geometry,—a necessary consequence of the complex character of the phenomena. It was, however, absolutely the only guardian standing between men and the effects of disease and accident.

There were dubious matters in every department of human activity, but in medicine and surgery that doubt assumed enormous importance, as human life was the issue. Uncertainty in diagnosis and treatment,—and every physician must at times be puzzled,—only increased the dignity of the profession, since the mental agony experienced by an honest, conscientious man under these conditions was greater than in any other pursuit. This sense of moral responsibility must be greater in the physician than in the pure scientist, lawyer, or even divine.

Dr. E. P. COOK, of Mendota, replied in appropriate terms to Governor Oglesby's address of welcome.

The President, Dr. D. S. BOOTH, delivered the usual annual address, under the title of

"HASH."

Dr. Booth discussed, in a humorous way, a variety of topics. At the ninth annual convention of the American Academy of Medicine, in Baltimore, 1884, a committee was appointed to memorialize Congress with reference to the establishment of a National Board of Medical Examiners.

Dr. D. S. Booth recommended the appointment of a similar committee from the Illinois State Medical Society, which should memorialize the State Legislature upon the same subject.

Female education, general and medical, was discussed at length. The germ theory of disease, the dangers of specialties, and sanitation received attention.

AFTERNOON SESSION.

Reports of General Committees.

GYNECOLOGY.—David Prince, Jacksonville; Sarah H. Stevenson, Chicago; O. B. Will, Peoria.

Dr. DAVID PRINCE, of Jacksonville, read a report on recent progress in gynecology, and exhibited a large number of instruments.

In the treatment of pelvic plastic exudates and uterine hyperplasia, he recommended, in addition to hot water, vaginal irrigation,—insisted on especially by Dr. Emmet,—the employment of the interrupted current.

OBSTETRICS.—C. W. Earle, Chicago; T. D. Washburn, Hillsboro; Anna S. Adams, Peoria.

Professor CHARLES WARRINGTON EARLE, of Chicago, read an interesting and valuable report on Obstetrics, in which allusion was made to the following topics:

At a meeting of physicians and surgeons in London, some years ago, an eminent member of the profession said, "Thank God! I know nothing of midwifery!" Modern science does not commend the insular arrogance of the remark.

ANATOMY AND PHYSIOLOGY.— Professor W. W. Jaggard, of Chicago, read a paper before the Obstetrical Section of the American Medical Association at its last annual meeting, in which recent progress upon the condition of the cervix during pregnancy and labour was sketched.

A gradual reversion to the views of the older obstetricians, Roederer and his pupil, Stein, Sr., had occurred within the past fifteen years. It was probable from the researches of Carl Braun (1857), Wilhelm Braune, Ludwig Bandl, Kuestner and Lahs, that during the latter months of pregnancy the supravaginal portion of the cervix is drawn up so as to form part of the general uterine cavity. While it is impossible to assert dogmatically that the contraction ring of Schroeder, the obstetrical internal os of Spiegelberg, the pelvic inlet stricture of Lahs, the ring of Bandl, the clinical or mechanical os of Ebell, are identical with the anatomical internal os, the weight of probable evidence is in favor of such a view.

THERAPEUTICS.— Professor Earle referred to the oxytocic properties of *Cimicifuga Racemosa*. (See CHICAGO MEDICAL JOURNAL AND EXAMINER, April, 1885, "*The Influence of Cimicifuga Racemosa on Parturition*," by Professor J. Suydam Knox); the external use of chloroform in labour; use of muriate of cocoaine in cases of rigid os, and perineum.

The value of electricity in the vomiting of pregnancy, post-partum hemorrhage, induction of premature labour, weak pains, was discussed at length, and the views of Dr Bater, of Texas, cited.

The bichloride of mercury had replaced carbolic acid among antiseptics, although there were cases of mercurial poisoning reported.

PATHOLOGY.—Since the time of Dr. C. W. Lever (1843), the association of albuminuria with puerperal eclampsia had been observed. Dr. Fry, of Washington, had recently discussed the pathology of the condition, and it was probable that eclampsia is not always due to uræmia. In addition to the other remedies, commonly employed, Professor Earle suggested *apocynum cannabinum*.

PRACTICE OF OBSTETRICS.—Occipito-posterior positions were more frequent than 'the older English text-books would lead one to believe. Leishman, in particular, was in error upon this subject.

Dr. Edward Warren Sawyer, of Chicago, had read a paper upon this topic before the American Gynecological Society, last October. Of Dr. Sawyer's forty-two cases, thirty-nine were right occipito-posterior, three left occipito-posterior; as to occurrence, one out of every five vertex presentations assumed this position.

Professor Earle recommended the manœuvre of Schatz, in the transformation of *face* into *vertex* presentations.

The old doctrine of the application of forceps in *breech* presentations had been revived. Professor Earle sanctioned the use of the instrument, under such conditions, believing it to be less harmful than the blunt hook.

Dr. Henry T. Byford's paper on "The Functions of the Membranes in Labour," (CHICAGO MEDICAL JOURNAL AND EXAMINER, March, 1885), deserved careful consideration. Dr. Byford was convinced of the possibility, probability and utility of preserving the membranes in labour until they dilate or aid in dilating the vulva.

In the immediate repair of a lacerated perineum, Professor Earle recommended the single suture method, suggested by Dr. Alloway, of Montreal.

In the treatment of retained membranes, in abortion, premature labour, or labour at term, there was great difference of opinion. Professor Earle would select a line of treatment which could not be termed meddlesome nor dilatory. He would not allow retention of the membranes to exist when they could be removed. Neither would he invert the uterus to peel off a shred of decidua. It was necessary to distinguish between simple retention of the separated placenta, on account of stricture of the internal os, and the placenta retained as the result of abnormal adhesion.

EXTRA-UTERINE PREGNANCY is of more frequent occurrence than was formerly believed. During the early months of pregnancy, Dr. Thomas's method of destroying the embryo by means of the interrupted current was the most rational mode of treatment.

When the full term of utero-gestation had expired, and the fœtus was living, laparotomy was indicated. When the term of gestation was prolonged, and the fœtus was dead, an expectant course of treatment should be pursued.

DISCUSSION.

Dr. DAVID PRINCE of Jacksonville, recommended the affusion of cold water upon the abdominal surface as the most efficient and least harmful method in the treatment of post-partum hemorrhage. He narrated the history of a case of post-partum hemorrhage, occurring in an eclamptic patient, successfully treated by this method.

Dr. C. TRUESDALE, of Rock Island, always advised the immediate operation for lacerated perineum. He did not recommend the single suture plan, as the surfaces ought to be very accurately approximated.

If this was not accomplished, pockets for the collection of pus and lochia would be formed, and the woman's condition would be more precarious than with the open wound.

Dr. WYNER, of Gilmore, always ruptured the membranes in labour when the cervix was fully dilated.

Dr. WENGER, of Gilman, was of the opinion that the configuration of the head could not be as favorable when the bag of waters was not ruptured. He always ruptured the sac of the amnion when the cervix was fully dilated.

Professor F. E. WAXHAM, of Chicago, had treated successfully over one hundred cases of retained placenta. He had used forceps and curette in but one case, and would not do so again. He removes, with his fingers, everything that can be removed; when decidual shreds remain within the uterine cavity, he irrigates twice daily with large quantities of carbolized water and exhibits ergot.

Dr. KREIDER, of Springfield, exhibited an improvised coil of rubber tubing, mounted on paste-board, to be used for mediate irrigation.

MATERIA MEDICA AND THERAPEUTICS—T. M. McIlvaine Peoria; J. F. Todd, Chicago; Catherine Miller, Lincoln.

Dr. T. M. McIlvaine, of Peoria, read a paper entitled "Medicinal and Non-Medicinal Therapeutics." Dr. McIlvaine disavowed any intention at plaigiarism. Dr. Austin Flint, Sr., had recently read a paper upon the same subject before the New York State Medical Association. Dr. McIlvaine referred to the optimists and pessimists in therapeutics. Between these extremes the body of the profession took its position. Therapeutics was derived from the Greek verb *therapeuo*, which meant to *wait upon* the sick or *alleviate* their sufferings, not to *cure*, or *drug* them. The lives of remedies were usually short, about twelve years. Statistics prove that the relative mortality in severe disease has not been diminished within the past twenty years. He narrated the history of a case of typhoid fever. The man could retain nothing, but demanded beer. The case was a desperate one, and his request was granted, and nine one-half gallons of beer were consumed in seven days, and the patient recovered.

A desperate case of pelvic cellulitis had recently come under his observation. The woman could not retain anything at all. Persistent nausea, vomiting and retching were so distressing symptoms that, in despair, he allowed her to eat sauer kraut. An uninterrupted convalescence began immediately.

The influence of faith in the treatment of disease received attention.

Dr. E. P. Cook, of Mendota, thought therapeutic agencies might be comprehended under three classes:

1. Positive Medication.
2. Rational Therapeutics.
3. Moonshine.

Drugs, with which positive medication could be effected, were few in number, and included the specifics. A rational line of treatment could be carried out with agents whose physiological action was not definitely known. Under this second class were comprehended by far the greatest number of therapeutic agencies. The third class, moonshine agencies, he would respectfully relegate to those transcendental philosophers, the homœopaths.

Professor N. S. DAVIS, of Chicago, thought there were few members of the medical profession who thought they could *cure* all diseases. Great injustice is done to the profession by indulgence in extravaganzas of this character. The statement of Dr. Chambers, that all disease was a retrograde movement, and that the patient must be supported throughout the condition, was an instance in point. Is this true? Exaltation and pathological increased functional activity demand sedatives. Depression and diminished functional activity demand stimulants. The nearer we keep to true representation, to the *truth*, the better for our own dignity, and the welfare of the world at large.

Therapeutics is the crowning glory of our profession. In the comprehension of this subject, a man cannot narrow down his vision to the condition of a single part. Attention had been diverted from the old, reliable methods of clinical observation to the microscopical study of disease. " Put salt on the microbe's tail, if you please, but don't kill the patient in the procedure." It is better for the microbe to kill the patient than for the physician to kill the patient in his hunt after the microbe.

In conclusion, Professor Davis said he only rose to check what he saw every day, namely, *a tendency to extravagance in every direction.*

Dr. T. F. WORRELL, of Bloomington, had grown up under the Anstie dispensation, and had been taught to employ not more than six or seven drugs. He had recently seen a prescription from a prominent physician, constructed on shot-gun principles, in which emetic, purgative, diaphoretic, diuretic, stomachic, tonic, alterative, and antiseptic properties or functions were combined.

"Is there a receiving agent, or distributing sentinel stationed in the stomach, who guards the ultimate fate of these products of polypharmacy?"

As a heart sedative, in pleurisy and pneumonia, he employed *veratrum viride;* as an emetic, ipecacuanha; as purgatives, rhubarb, calomel, senna, blue mass.

The incorporation of strychnine in a pill for constipation, on the theory that it increased peristalsis, was absurd. Bleeding, an invaluable resource, under certain conditions, had been relegated to the obsolete procedures of medical art.

Professor F. E. WAXHAM, of Chicago, never prescribed a remedy without a perfectly distinct reason for its exhibition.

Dr. E. P. COOK, of Mendota, desired to protest against the statement that polypharmacy was an outgrowth of the modern school of therapeutics.

Dr. A. WETMORE, of Waterloo, desired to enter a plea for *mistura composita.* A prescription, of the kind to which Dr. Worrell alluded, had its paradigm in the food we eat.

OPHTHALMOLOGY AND OTOLOGY.—W. T. Montgomery, Chicago; C. R. Park, Bloomington; Robert Tilley, Chicago.

Professor W. T. MONTGOMERY, of Chicago, read a report on *Ophthalmology* and *Otology*, in which allusion was made to the following topics:

JEQUERITY.—Two years ago, Professor Montgomery had visited De Wecker's eye clinic in Paris and learned the use of jequerity.

Jequerity was to chronic granular conjunctivitis what irridectomy was to glaucoma. In the Illinois Charitable Eye and Ear infirmary, Professor Montgomery had treated about two hundred and fifty eyes, in all forms of the disease, except the muco-purulent variety with non-vascular cornea.

A three per cent. infusion was the most eligible preparation. As to method, after eversion of the eye-lids, a brush saturated with the infusion, was passed over the surfaces twelve times. Within twenty-four hours, the desired inflammation, a membranous exudate, was produced. The local disturbance, pain and swelling, was usually slight. The constitutional effects were usually trivial, although chill and fever had in some cases followed. The acme of the inflammation was usually reached at the end of the second day. During this period, the eyes could be bathed with cold water and some anodyne. Usually two applications are required to cure a case.

His results with the drug may be tabulated as follows:

10 % cured by one application.
25 % " " two or three applications.
30 % " " three to fifteen applications.
20 % improved by one application.
20 % not improved at all.

In no case could the inflammation be evoked beyond the fourth time. In only two cases could it be produced three times.

It required one to three weeks for recovery.

He had never seen sloughing of the cornea follow the application, although he had seen three cases of dacryocystitis.

He regarded the drug as almost a specific in chronic granular conjunctivitis with vascular cornea, especially in hard granulations with pannus.

It was not a dangerous remedy, when used with the following precautions:

I. Never employ when the cornea is clear.

II. Repeat only at intervals of twenty-four hours.

III. Permit any inflammation to subside before the second application.

The anæsthetic effect of *Cocoaine* was the most remarkable discovery made since that of general anæsthesia.

Since Nov. 1st, 1884, he had employed that remedy in the following operations:

Cataract, two; needle operations, fifteen; iridectomy, five; iridectomy for glaucoma, two; foreign body in anterior chamber, one; tenotomy, thirty; foreign body in cornea, Bowman's operation for pterygium.

He employed a two to four per cent. solution.

The action of the remedy was not perfectly satisfactory in iridectomy for glaucoma, or in the removal of a foreign body from the anterior chamber. In tenotomy of the *recti*, after injection of a few drops of the solution into the cellular tissue, no pain was complained of.

Ten cases of phlyctenular keratitis, with good results had been treated. In twelve cases of iritis, the relief of pain and other symptoms was only partial. One case of episcleritis was cured by conjoined use of iodide of potassium, bichloride of mercury and cocoaine. As the man had a specific history, it was impossible to tell whether or no cocoaine had effected the cure.

Professor Alfred Graefe's operation, entitled, *Evisceratio bulbi*, as a substitution for enucleation, had two advantages, (1) the danger from meningitis was less; (2) the stump is superior. Professor E. L. Holmes, of Chicago, had for years been performing the same operation, which Graefe claims as original.

In 1876, Professor Montgomery himself had operated by Professor Holmes's method.

CHANCRE OF EYE-LID PRODUCED IN AN UNUSUAL WAY.—Professor Montgomery narrated the history of a case of chancre of the eye-lid produced by the insertion of the tongue under the palpebral conjunctiva, in an effort to remove a foreign body.

DISCUSSION.

Dr. MENDENHALL, of Georgetown, thought hot applications in chronic granular conjunctivitis better than cold ones.

Dr. J. D. SKEER, of Chicago, wished to inquire whether there was any violent reaction after the use of cocoaine. Does the solution deteriorate with time? He had been told that it was impossible to keep the solution longer than three weeks.

Professor SAMUEL J. JONES, of Chicago, thought that in the employment of jequerity, ophthalmologists were running an unnecessary risk. The local disturbance and constitutional reaction were not trivial facts. To get rid of the opacity, ulceration was produced. This ulceration could not always be arrested when desired, and sloughing of the cornea had resulted. At present, this method of treatment is empirical. We have safe remedies, by which the same result can be secured. A time may come when we shall know more of the physiological action of jequerity, and when it will be employed as a safe and valuable remedy. The patient ought not to be subjected to greater risks than the physician, himself, would assume.

De Wecker secured the drug from a South American patient, and rushed into print after a very inadequate experience with the remedy.

The hydrochlorate of cocoaine was useful in the production of a superficial anæsthesia. In two cases of iridectomy, in his own practice, the drug did not annul all pain. Where general anæsthesia was contra-indicated, the drug would prove invaluable. So much had been claimed in favor of the remedy that he feared reaction, which might prevent its being given the credit to which it is justly entitled.

Professor Montgomery, in reply to Dr. Skeer, said that he had observed no change in the action of his cocoaine solution, and it was three months old. In reply to Professor Jones, he thought that in the treatment of his own case, he would use jequerity.

Adjourned.

*Second Day—Wednesday, May 20th.*

The Society was called to order by the President, Dr. D. S. BOOTH, of Sparta.

Dr. Prewett, Dr. Dickinson, and Dr. Middlecamp, delegates from the Missouri State Medical Society, were introduced.

Dr. JOHN H. RAUCH, Secretary of the State Board of Health, read a report on Medical Legislation. Dr. Rauch called attention to the bills affecting medical interests, now pending in the Senate and House.

Four members of the Senate and eight members of the House were members of the regular profession.

Dr. A. B. STRONG, President of the Demonstrators' Association of Chicago, made a report on the Anatomy bill. The bill had passed its third reading in both Senate and House. It would probably become a law. Three thousand medical men had responded to communications from the Demonstrators' Association. Every one was thoroughly in earnest.

At a later period, he desired to call attention to the law regulating malpractice suits, but thought all energies, for the present, should be concentrated upon the Anatomy bill.

Dr. D. A. SHEFFIELD, member of the House from Jo Daviess Co., stated that bill No. 73, known as the "Anatomical Bill," would probably become a law, but urged every member of the Society to confer with his representatives upon the subject.

Dr. SHUMWAY, Dr. WHITE, Dr. HANNA, Dr. WEIR, Dr. MILAM, representatives, were in favor of the bill, and hoped to see it become a law in a few days.

Dr. E. P. COOK, of Mendota, was glad to see so much healthful enthusiasm upon these subjects of so great medical interest.

COMMITTEE ON LEGISLATION FOR THE INSANE.—B. M. Griffith, Springfield, Chairman; Walter Hay, Chicago; J. L. White, Bloomington; F. B. Haller, Vandalia; W. A. Haskell, Alton; E. P. Cook, Mendota; Wm. Hill, Bloomington; A. B. Strong, Chicago.

Professor WALTER HAY, of Chicago, reported that the "Campbell Bill" had already passed its third reading, and he thought it would become a law.

Dr. J. M. FOWLER, member of the House from the fifty-first district, thought the prospect for the success of the Campbell bill was excellent.

NECROLOGY.—E. Ingals, Chicago; William Hill, Bloomington; M. F. Bassett, Quincy.

Dr. M. F. BASSETT, of Quincy, reported one death.

Abner Hard, M. D., of Aurora, was born in New York, 1820; when eleven years old he came to Michigan; in 1884 he took up his residence in Missouri; in 1847 he settled in Aurora. He was graduated at the Keokuk Medical College in 1851, and died March 20th, 1885. He was Surgeon to the Eighth Regiment of Illinois Cavalry, served in every battle from Malvern Hill to Gettysburg, and in 1862 was breveted Lieutenant-Colonel. He was President of the Fox River Medical Association. He died in the line of duty, from blood-poisoning, contracted during an operation.

A recess of ten minutes was given for the selection of a Nominating Committee.

The following Special Committees were then called:

LARYNGOLOGY.—E. F. Ingals, Chicago.

Professor E. F. INGALS, of Chicago, read a paper entitled:

"Recurrent Laryngitis in Relation to Obstruction of the Nares, or Chronic Catarrh."

Dr. J. P. MATTHEWS, of Carlinville, read the history of the following case of tracheotomy for laryngeal obstruction, and exhibited the patient.

The patient, twenty-eight years old, had a good antecedent history. In October, 1884, he caught cold and became hoarse. Dr. Matthews attended him in November, when he had a severe attack of scarlet fever, which lasted thirty days. Hoarseness subsided about the thirtieth day. Dyspnœa increased. The laryngoscope revealed thickening of the arytaeno-epiglottidean folds, and stenosis of the larynx. Bloody pus was expectorated and, in the absence of all pulmonary complications, led to the diagnosis of ulcer, immediately below the vocal cords.

On January 6th, 1885, tracheotomy was performed. A large quantity of bloody pus was expectorated, and the diagnosis of ulcer was confirmed. The dyspnœa was relieved at once. Dr. Matthews thinks the tube can now be removed with safety. He referred incidentally to a case of spasmodic asthma, cured by the treatment of a post-nasal catarrh. The ulcer in the first case was due to typhoid fever.

DISCUSSION.

Dr. PREWETT, of St. Louis, had treated certain cases of obstruction of the posterior nares by breaking off a bit of the septum with Adams' forceps.

He had performed tracheotomy for sudden dyspnœa in a case of pneumonia. A fibrinous cast was expelled from the trachea and the patient recovered.

Professor INGALS thought that laryngotomy must be performed in Dr. Matthews' case.

Professor F. E. WAXHAM, of Chicago, referred to the advantage of intubation of the larynx in cases of stenosis.

PEDIATRICS.—A. F. Rooney, Quincy.

In the absence of Dr. A. F. Rooney, chairman of the Committee, Professor A. E. HOADLEY, of Chicago, read a paper entitled, "Too often Feeding as a Cause of Disease in Infants, and a Protest against one Cow's Milk." The paper was discussed by Dr. Henry T. Byford, of Chicago, Dr. Middlecamp, of Kansas City, Professor F. E. Waxham, of Chicago, and Dr. Milam.

DERMATOLOGY.—W. J. Maynard, Chicago.

Owing to the absence of Professor W. J. Maynard, of Chicago, chairman of the Committee, Professor H. J. REYNOLDS, of Chicago, read a paper, entitled "The Treatment of Eczema." The paper appears in another column of the JOURNAL.

TETANUS.—C. Truesdale, Rock Island.

Dr. C. TRUESDALE of Rock Island, read a report on Tetanus. Dr. Truesdale had recently treated two cases of tetanus by the hypodermic injection of a six per cent. solution of carbolic acid into the cellular tissue of the injured part, and the internal exhibition of morphia, calabar bean, chloral and bromide of potassium, pushed to their full physiological action, and administered in succession. Dr. G. G. Craig, Health Commissioner of Rock Island, had treated two cases in the manner suggested by Dr. Truesdale. The four cases recovered.

INSANITY.—R. J. Patterson, Batavia.

Dr. R. J. PATTERSON, of Batavia, read a report on Insanity. There are in the United States about 100,000 insane persons, a little less than one-half of whom are in hospitals, and a little more than one-half in county alms-houses, jails, and in private families. It is believed that the insane are increasing somewhat beyond the proportionate increase in population. In view of these facts, it is well to renew the inquiry, if more may not be done than has been accomplished through medical influence, in certain directions, toward the prevention of insanity, in cutting off some of its feeders and drying up some of its sources. It is believed that the family physician should be, and is destined to be, more and more consulted in regard to questions in connection with hereditary influences, and although the questions involved are intensely personal, his opinions, as physician and friend, may sometimes prevent or lessen the *propagation* of those degenerate conditions, and the incurable diseases upon which insanity often depends.

Attention was then called to certain cases of insanity that were believed to be most amenable to treatment at home.

ALCOHOL AS A THERAPEUTIC AGENT.— M. F. Bassett, Quincy.

Dr. M. F. BASSETT, of Quincy, in his report upon alcohol as a therapeutic agent, said that its therapeutic use should be restricted to the four conditions of *shock*, *collapse*, *septicæmia* and *snake-bites*. Even when used under these circumstances, it might be replaced by other, quite as efficient agents. The danger of the formation of the alcohol habit was so great that it should outweigh, in many cases, the direct benefit to be gained.

DISCUSSION.

Dr. E. P. COOK, of Mendota, said that while he deprecated the evil results which sometimes follow the administration of stimulants, yet he desired to make a protest against the reading of temperance homilies before a scientific body of physicians.

Dr. T. F. WORRELL, of Bloomington, endorsed every word Dr. Bassett had uttered.

PHYSIOLOGY.—A. Wetmore, Waterloo.

Dr. A. WETMORE, of Waterloo, read a brief report on recent progress in vegetable physiology.

TREATMENT OF EPILEPSY.—D. R. Brower, Chicago.

Professor D. R. BROWER, of Chicago, drew the following conclusions in his report on epilepsy:

I. Surgical procedures offer the most certain methods of cure,—the removal of cicatrices, trepanation, oöphorectomy and ligation of the vertebral arteries, being the most important.

II. The medical treatment should be mainly addressed to building up the general nutrition of the patients,—brucia, strychnia and arsenic being the most valuable therapeutic agents.

III. Depressing agents, such as the bromides, are palliative rather than curative. To be curative they should be pushed to bromism, hoping thereby to so alter the nutrition of the nerve centers as to make the epileptic habit impossible, when they are withdrawn.

ON THE INFLUENCE OF APPRECIABLE METEOROLOGICAL AND TOPOGRAPHICAL CONDITIONS ON THE PREVALENCE OF ACUTE DISEASES.—N. S. Davis, Chairman, Chicago; J. H. Hollister, Chicago; J. F. Todd, Chicago; E. P. Cook, Mendota; G. W. Jones, Danville.

Professor N. S. DAVIS, in his report, said that stations and observers throughout the State had been selected, and instruments of precision distributed. No facts of importance had accumulated as yet, as the work had only been completed within the past year.

On motion, the report was accepted, and the committee continued.

Dr. H. R. GUTHRIE, of Sparta, read a paper on medical education.

The continuation of the paper was referred to the first order of business on Thursday morning.

*Third Day—Thursday, May 21st.*

MORNING SESSION.

The society was called to order by the President. Dr. D. S. BOOTH, of Sparta.

Dr. Guthrie completed the reading of his paper.

Dr. G. N. KREIDER, of Springfield, read a paper on medical education, in which he complimented the Direction of Cornell University on the excellent preliminary course to the study of medicine.

DISCUSSION.

A lively debate on the papers of Drs. Guthrie and Kreider ensued, in which Dr. Milam, Dr. H. T. Byford, Dr. A. Wetmore, Professor C. W. Earle, Dr. Myers. Dr. Wenger, Dr. Remsburg, Dr. Veatch, Professor F. E. Waxham, took part.

The following General Committees were then called:

PRACTICAL MEDICINE.—Norman Bridge, Chicago; C. H. Norred, Lincoln; J. R. Livingood, Rossville.

Owing to the absence of Professor NORMAN BRIDGE, of Chicago, a brief abstract of his report was read. The following topics were discussed:

1. Intubation of the Larynx, with exhibition of instruments.
2. Fever viewed as a physiological process.
3. Cholera,—prophylaxis.
4. Trypsin as a solvent of croupous or diphtheritic exudate.
5. Treatment of Typhoid Fever.
6. Inhalation of defibrinated blood.
7. Hypodermatic injection of human blood.

Dr. C. H. NORRED, of Lincoln, read a supplementary report on the Practice of Medicine, embodying a stenographic report of the evidence in the Burns-Carpenter case.

Dr. Norred claimed that experts can determine, by post-mortem signs, the probable time of death. Temperature of the cadaver is not so important a sign as *rigor mortis.*

SURGERY.—W. A. Byrd, Quincy; D. A. K. Steele, Chicago; Cass Chenowith, Decatur.

Dr. W. A. Byrd, of Quincy, read a report on Surgery. After referring to recent contributions to surgical literature during the past year, he called attention to a new tourniquet, applicable in cases of hip-joint amputation. He narrated the histories of two cases, in which the tourniquet was employed with a minimal loss of blood.

The Society adjourned at 12:30 P. M.

Report of the Nominating Committee.

*President.*—W. A. Byrd, of Quincy.

*First Vice-President.*—W. T. Kirk, of Atlanta.

*Second Vice-President.*—A. Wetmore, of Waterloo.

*Permanent Secretary.*—S. J. Jones, of Chicago.

*Treasurer.*—Walter Hay, of Chicago.

*Judicial Committee.*—E. Ingals, of Chicago; F. B. Haller, Vandalia; W. Hill, Bloomington.

*Assistant Secretary.*—Hiram Luce, Bloomington.

*Members of Committee of Arrangements.*—J. L. White, T. F. Worrell, Lee Smith, A. T. Barnes, Wm. Hill, of Bloomington.

GENERAL COMMITTEES.

*Practice of Medicine.*— M. F. Bassett, Quincy; J. J. M. Angear, Chicago; H. R. Guthrie, Sparta.

*Surgery.*—C. Truesdale, Rock Island; T. M. McIlvaine, Peoria; M. Reece, Abingdon.

*Obstetrics.*—W. W. Jaggard, Chicago; Hugo Rothstein, Waterloo; E. A. Ingersoll, Canton.

*Gynecology.*—J. H. Etheridge, Chicago; M. J. Mergler, Chicago; C. C. Hunt, Dixon.

*Drugs and Medicines.*—E. B. Montgomery, Quincy; J. M. G. Carter, Waukegan; Katharine Miller, Lincoln.

*Ophthalmology and Otology.*—Robert Tilley, Chicago; A. C. Carr, Carlinville; W. T. Speaker, Mt. Morris.

*Necrology.*—E. Ingals, Chicago; Wm. Hill, Bloomington; G. W. Cox, Clayton.

SPECIAL COMMITTEES.

*Orthopedic Surgery.*—C. E. Webster, Chicago.

*Diseases of Children.*—F. E. Waxham, Chicago; C. B. Slater, Aurora.

*Microscope in Gynecology.*—O. B. Will, Peoria.

*Dermatology.*— H. J. Reynolds, Chicago; W. J. Maynard, Chicago.

*Physiology.*—A. Wetmore, Waterloo.

*Insanity.*—A. T. Barnes, Bloomington.

*Committee on Legislation for the Insane.*—Walter Hay, Chicago; E. P. Cook, Mendota; F. B. Haller, Vandalia.

*Medical and Sanitary Legislation.*—B. M. Griffith, Springfield; M. W. Seaman, Shipman; J. L. White, Bloomington; W. A. Haskell, Alton; A. B. Strong, Chicago.

*Biographical Committee.*—J. H. Hollister, Chairman, Chicago; E. P. Cook, Mendota; E. Ingals, Chicago; J. T. Curtis, Otterville; D. E. Foote, Belvidere; George W. Jones, Danville; F. B. Haller, Vandalia; T. F. Worrell, Bloomington; Washington West, Belleville; Robert Boal, Peoria.

*On the Influence of Appreciable Meteorological and Topographical Conditions on the Prevalence of Acute Diseases.*—N. S.

Davis, Chairman, Chicago; J. H. Hollister, Chicago; J. F. Todd, Chicago; E. P. Cook, Mendota; G. W. Jones, Danville.

*Committee on Publication.*—J. F. Todd, Chairman, Chicago.

Place of next meeting—Bloomington, Ill.

Day of next meeting—Third Tuesday in May, 1886.

The report of the nominating committee was adopted.

